# Experimental observation of anomalous topological edge modes in a slowly driven photonic lattice

**DOI:** 10.1038/ncomms13918

**Published:** 2017-01-04

**Authors:** Sebabrata Mukherjee, Alexander Spracklen, Manuel Valiente, Erika Andersson, Patrik Öhberg, Nathan Goldman, Robert R. Thomson

**Affiliations:** 1 Scottish Universities Physics Alliance (SUPA), Institute of Photonics and Quantum Sciences (IPaQS), Heriot-Watt University, Edinburgh EH14 4AS, UK; 2 Center for Nonlinear Phenomena and Complex Systems, Université Libre de Bruxelles, CP 231, Campus Plaine, B-1050 Brussels, Belgium

## Abstract

Topological quantum matter can be realized by subjecting engineered systems to time-periodic modulations. In analogy with static systems, periodically driven quantum matter can be topologically classified by topological invariants, whose non-zero value guarantees the presence of robust edge modes. In the high-frequency limit of the drive, topology is described by standard topological invariants, such as Chern numbers. Away from this limit, these topological numbers become irrelevant, and novel topological invariants must be introduced to capture topological edge transport. The corresponding edge modes were coined anomalous topological edge modes, to highlight their intriguing origin. Here we demonstrate the experimental observation of these topological edge modes in a 2D photonic lattice, where these propagating edge states are shown to coexist with a quasi-localized bulk. Our work opens an exciting route for the exploration of topological physics in time-modulated systems operating away from the high-frequency regime.

## Abstract (web summary)

A periodically driven system may show a novel type of topologically protected edge modes that has no static analogue. Here, Mukherjee *et al*. report the observation of such anomalous chiral edge modes in an ultrafast-laser-inscribed slowly-driven photonic lattice.

The discovery of the quantized Hall effect, and its subsequent topological explanation, demonstrated the important role topology can play in determining the properties of quantum systems^1^,^2^,^3^. This realization led to the development of topological band theory, where, in addition to band index and quasimomentum, Bloch bands are also characterized by a set of topological invariants. This topological theory can be readily extended to periodically driven systems. In the limit of fast driving, the topology of the system can still be captured by the topological invariants used to describe static systems^4^,^5^. Away from this high-frequency regime, however, situations can arise where standard topological invariants are zero, but yet, topologically protected edge modes are still observed^4^,^6^,^7^,^8^,^9^. These anomalous topological edge modes have no static analogue, and are associated with a distinct topological invariant, which takes into account the full time evolution over a driving period.

Subjecting a system to time-periodic modulations constitutes a powerful method to engineer band structures with non-trivial topological properties^4^,^5^,^10^,^11^,^12^,^13^, as recently demonstrated in cold-atom experiments^14^,^15^,^16^ and photonics^17^,^18^. In this Floquet-engineering approach, a static system, described by a Hamiltonian 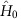, is driven periodically in time by a modulation 
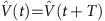
, the frequency of which shall be denoted by *ω*=2*π*/*T*. The time-evolution operator over an arbitrary long duration Δ_*t*_=*t*_f_−*t*_0_ then takes the general form^12^,^19^


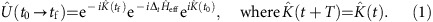


Here the time-independent (effective) Hamiltonian 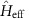 describes the time-averaged dynamics over the duration Δ_*t*_, which can be isolated by probing the system stroboscopically at discrete times Δ_*t*_=*T* × integer. This effective Hamiltonian stems from a rich interplay between the static system 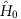 and the time modulation 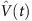 (refs 12, 13); its band structure (the quasienergies or Floquet spectrum) can feature topological properties, such as non-zero Chern numbers and chiral edge modes^4^,^5^,^10^,^11^,^12^,^13^, in direct analogy with non-driven systems. The time-evolution operator (equation (1)) also includes the micro-motion operator 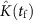, which describes the dynamics that takes place within each period of the driving, that is, when Δ_*t*_≠*T* × integer. In the high-frequency regime of the driving (*ω*→∞), observables are often only slightly affected by the micro-motion. In this regime, the topological properties of the driven system are then entirely captured by the effective Hamiltonian 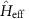. In particular, the observation of topological edge modes can be directly related to the Chern numbers associated with the effective band structure^4^,^5^. Such edge modes were directly visualized in photonic realizations of Floquet band structures^17^ (see also refs 20, 21, 22, 23 for other photonic implementations of standard topological edge modes). Importantly, this is no longer the case away from the high-frequency regime, when *ħω* becomes comparable to the typical energy scales of the system (for example, the bandwidth of the effective band structure^4^,^6^,^7^,^8^,^9^,^24^,^25^,^26^). In this case, the micro-motion strongly affects the time evolution of observables, and the topological properties of the system can no longer be simply related to the effective Hamiltonian only. In particular, the presence of topological edge modes is now entirely ruled by a distinct topological invariant, a winding number *W* that takes into account the micro-motion^6^,^7^. As realized in refs 4, 6, this allows for the presence of topological edge modes even when the Floquet bands resulting from 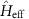 are all associated with zero Chern numbers: this singular situation defines the so-called anomalous regime^6^, where the presence of topological edge modes stems from the micro-motion, and not from time-averaged dynamics only. This intriguing regime of periodically driven systems has recently been investigated in a diverse range of experimental platforms: topologically protected bound states associated with non-trivial *W* were first demonstrated in a one-dimensional photonic set-up realizing a discrete time quantum walk^27^; more recently, ref. 28 reported on the observation of anomalous edge modes in a designer surface plasmon platform. Finally, ref. 29 considered a Thouless-pump approach to measure *W* in a one-dimensional microwave network.

Here we demonstrate the first experimental observation of anomalous topological edge modes in an ultrafast-laser-inscribed photonic lattice. This fabrication technique enables the realization of a photonic lattice where each bond is addressed independently and dynamically, generating a rich band structure with robust anomalous chiral edge modes and the potential for perfectly localized bulk states.

## Results

### Theoretical background

**Figure 1:**
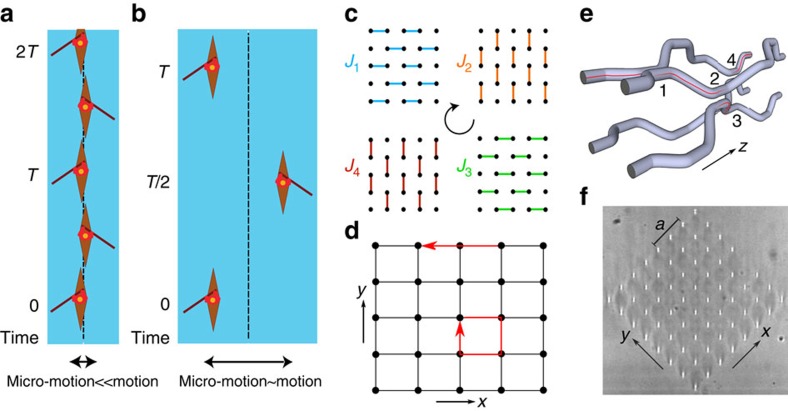
Slowly driven systems and photonic implementation. Sketch illustrating the high-frequency regime (**a**), and away from the high-frequency regime (**b**), for a simple driven system, here represented by a rower. In the high-frequency regime, the micro-motion typically only slightly affects the motion: the time evolution is well captured by time-averaged dynamics, which can be probed stroboscopically at discrete times Δ_*t*_=*T* × integer. Away from the high-frequency regime, the micro-motion can significantly affect the dynamics, and the time evolution is no longer well described by the time-averaged Hamiltonian only. (**c**) The four different bonds present in the lattice (with coupling constants *J*_1,2,3,4_) and the cyclic driving protocol employed. (**d**) In the simplest case where all bond strengths are equal, *JT*/4=*π*/2, chiral edge modes arise, while the bulk is localized (the evolution operator over one period of the drive is trivial (identity) in the bulk). (**e**) Sketch illustrating how, using ultrafast laser inscription, different pairs of waveguides can be moved together to turn on a coupling and then apart to switch it off. Within the paraxial approximation, this flexibility allows for a realization of the driving protocol shown in **c**. In waveguide arrays, the propagation direction *z* plays a role analogous to time^36^. (**f**) White-light transmission micrograph of the facet of the laser-fabricated photonic lattice. Note that the axes of the photonic lattice were rotated by 45° with respect to the vertical to obtain equal coupling constants along the two axes (ref. 37). Here *a* is the lattice constant.

The theory works in refs 4, 6 introduced conceptually simple models that exhibited such intriguing Floquet topological structures, which appear away from the high-frequency limit (Fig. 1a,b). The model in ref. 6 is a square lattice with nearest-neighbour couplings, which are engineered so that the couplings between a lattice site and its four nearest neighbours are independently controllable. These four couplings, denoted *J*_1_ through *J*_4_, are then varied in a spatially homogeneous and time-periodic manner so that any lattice site is, at any given moment, coupled to only one of its nearest neighbours (Fig. 1c). The simplest demonstration of this model is when the driving period *T* is split into four equal steps, and *T* is selected such that a particle that is initially localized on a certain site will be completely transferred to the neighbouring site after a time *T*/4. Therefore, considering for now a system without edges, this means that after one complete period, any initial state is exactly reproduced, that is, the propagator, 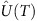, is the identity matrix (Fig. 1d). As a corollary, the effective Hamiltonian in equation (1) is the zero matrix and the Floquet spectrum consists of two degenerate flat bands at zero energy. The bulk is completely localized, and the Chern numbers associated with the effective Hamiltonian are necessarily trivial. In a geometry with edges, however, it is found that there are chiral propagating edge modes that are localized along the edge. These occur because a particle launched at an edge cannot return to its initial position and instead move one unit cell along the edge (Fig. 1d), in direct analogy with the skipped cyclotron orbits of quantum Hall systems.

As previously mentioned, the topological properties of slowly driven systems are well captured by a winding number that takes the full time evolution into account, including the micro-motion^6^,^7^. For the two-band model introduced above, the time-evolution operator at time *t*, where 0≤*t*<*T*, may be written as


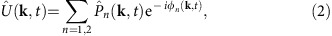


where 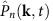 and 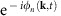 are the projectors onto the eigenstates and the corresponding eigenvalues of 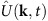, respectively. The instantaneous energies, *ϕ*_*n*_/*T*, are defined modulo 2*π*/*T*, and we define the corresponding Floquet–Brillouin zone to be in the range [−*π*/*T*, *π*/*T*]. In a driven system, there are two types of degeneracy that can occur within a driving period: inter-zone degeneracy, where *ϕ*_1_(**k**, *t*)=*ϕ*_2_(**k**, *t*), and degeneracy through the zone edge, where *ϕ*_1_(**k**, *t*)=−*π* and *ϕ*_2_(**k**, *t*)=*π*. It is the existence of this latter type of degeneracy that profoundly alters the topological picture of driven systems. Zone-edge singularities allow the Chern number of the bands to change without the inter-zone gap closing. Consequently, these zone-edge degeneracies can lead to violations of the static bulk-edge correspondence^30^, so that the Chern number of both Floquet bands are zero but yet protected edge modes are still present^4^. The adequate topological characterization of driven systems is captured by a winding number that includes the changes in Chern numbers that occur through the zone edge, within a period of the driving. In a two-band driven system, there can be at most two bandgaps, and for each of these bandgaps, there is a winding number *W*_*m*_, which has the form^7^


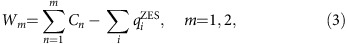


where *C*_*n*_ is the Chern number of the *n*th band at time *T*, and where *q*_*i*_ corresponds to the change in Chern number of the lowest band that occurs in the *i*th zone-edge degeneracy. This winding number directly gives the number, *n*_edge_(*m*), of topologically protected chiral edge modes present in the *m*th gap. This bulk-edge correspondence for driven systems can be shown to have the form^7^


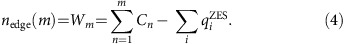


The first term in the second equality of equation (4) is the term that is found in static systems, while the second applies only to driven systems. It is this term that is the source of the anomalous edge modes analysed in this work, as it allows the number of edge modes to be non-zero even if the standard topological invariants, that is, the Chern numbers *C*_*n*_, are zero for all of the Floquet bands.

### Proposed driving protocol

**Figure 2:**
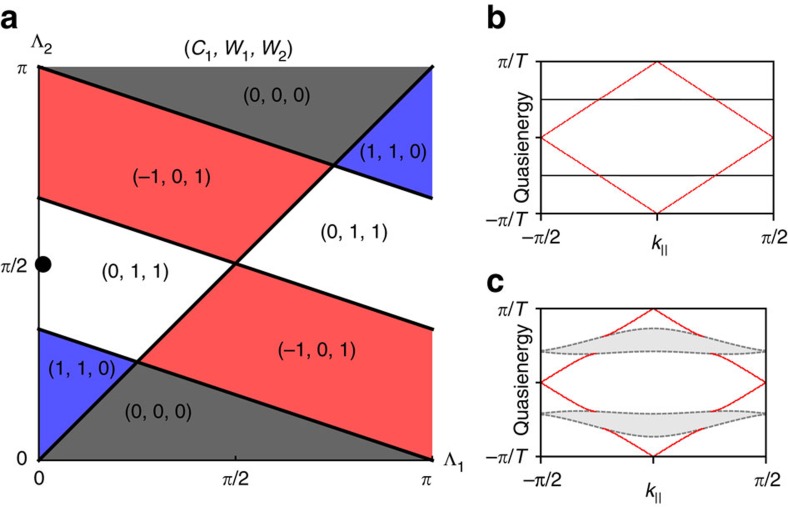
Topological phase diagram and band structures. (**a**) Phase diagram for a generalization of the model in Fig. 1c, which allows the bond *J*_1_ to be of a different strength to the bonds 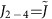. Here Λ_1_=*J*_1_*T*/4 and Λ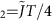. For each topological phase, we indicate three topological invariants: the Chern number of the lowest Floquet band *C*_1_, the winding number *W*_1_ of the gap centred around zero, and the winding number *W*_2_ of the gap centred around *π*/*T*. The white regions indicate where anomalous edge modes can be observed (*C*_1_=0, *W*_1,2_≠0). Note that *C*_1_=*W*_1_−*W*_2_. (**b**) Quasienergy spectrum for the parameters indicated by the black dot in **a**. The spectrum was calculated for a strip-geometry aligned along the *x* direction, and closed along the *y* direction (Fig. 1d). Red curves indicate topological-edge modes dispersions, while black curves correspond to the bulk bands. We note that the edge-modes velocity depends on the orientation of the strip (not shown here). (**c**) Quasienergy spectrum for the experimentally achieved parameters. The first Floquet–Brillouin zone is taken to be defined in the range [−*π*/*T*, *π*/*T*].

In this work, we experimentally implement a variant of the model introduced in ref. 6. In our model, the coupling of the first bond (*J*_1_) is different than the following three bonds (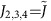), which are chosen so as to satisfy *J*_1_*T*/4=Λ_1_ and 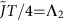. This gives two controllable experimental parameters, Λ_1_ and Λ_2_, that can be tuned independently. We find that the interplay between these two parameters produces a rich phase diagram, shown in Fig. 2a, which demonstrates how this simple model can be used to explore many different topological regimes (see Methods). Each topological phase of this driven two-band system is accurately labelled by the winding numbers associated with the two bandgaps; in the phase diagram shown in Fig. 2a, we have chosen *W*_1_ to be the winding number for the bandgap centred around quasienergy zero and *W*_2_ for the bandgap centred around *π*/*T*. In addition, we also provide the Chern number of the lowest Floquet band *C*_1_, so as to highlight the aforementioned anomalous regimes, where *C*_1_=0 and *W*_1,2_≠0.

As proposed in ref. 6, the simplest way to experimentally demonstrate the existence of anomalous edge modes is to choose the isotropic configuration Λ_1_=Λ_2_=*π*/2. However, in this case, the Floquet bulk bands are degenerate at *ɛ*=0, and an arbitrary small variation in the parameters value can potentially drive the system out from the anomalous regime; see the central point (Λ_1_=Λ_2_=*π*/2) of the phase diagram displayed in Fig. 2a. Since such variations are expected to be present in our experimental set-up, due to limited but unavoidable disorder, an unambiguous realization of anomalous edge modes requires instead to work deep within the anomalous regimes demarcated in Fig. 2a. For the sake of experimental practicability, we aim to focus on the anomalous edge modes that are predicted for Λ_1_=0 and Λ_2_=*π*/2, as indicated by the black dot in Fig. 2a. In this configuration, the blue bonds in Fig. 1c are never turned on, while complete transfer of light can occur via the other three bonds. These parameters are desirable as not only is the resultant system located well within the anomalous regime (Fig. 2a) but also the associated spectrum consists of two gapped flat bands (Fig. 2b). In addition, the Floquet states have a simple analytical form, and there are robust chiral edges states that can be excited with unit efficiency, through the simple experimental technique of single-site excitation.

### Photonic implementation

The realization of this model requires a high degree of control over the couplings present in the lattice. Ultrafast-laser-inscribed arrays of optical waveguides with three-dimensional geometry offer this control, as they allow the coupling between lattice sites to be individually controlled; see the sketch in Fig. 1e for a general situation with *J*_1,2,3,4_≠0 and Fig. 1f for an image (cross-section) of the actual experimental set-up. In the experiment presented here, a lattice constant of 40 μm was chosen, such that the coupling between lattice sites was insignificant over the maximum observable propagation distance. To turn on the coupling between any two waveguides, the inter-waveguide separation is reduced such that the two waveguides propagate together for a 4.5 mm straight section, with a centre-to-centre waveguide separation of 11 μm. After this interaction region, the waveguides again separate in a reverse manner. For a particular wavelength, the coupling that occurs between two synchronously bending waveguides can be shown to be equivalent to that of two straight waveguides with some effective coupling constant (Supplementary Note 1). This effective coupling can be controlled by changing the interaction length and/or the separation of the waveguides; these parameters are fixed upon writing the sample and so do not allow fine-tuning of the coupling *in situ*. However, the wavelength of the light used to excite the lattice is a tunable parameter that can be used to control the effective coupling strength (see Methods). To demonstrate the existence of anomalous edge modes, a lattice of 63 sites, consisting of two driving cycles, was fabricated. This lattice contains a single defect, namely, a missing lattice site on the edge, which allows one to verify that the edge modes indeed freely propagate around the system, irrespective of its shape. It should be mentioned that the propagation of light waves along the propagation direction (*z*) of the photonic lattice mimics the time evolution of a particle moving in a tight-binding lattice, within the paraxial approximation.

To characterize the bonds present in the lattice and validate our theoretical model, we fabricated five sets of each bond in isolation, inside the same substrate and measured their behaviour as a function of the wavelength of light (Supplementary Note 2). When characterized with 785 nm light, the mean and standard deviation (s.d.) of Λ_*i*_ (*i*=2, 3, 4) were found to be Λ_2_=*π*/2(1.16±0.04), Λ_3_=*π*/2(1.15±0.04) and Λ_4_=*π*/2(0.85±0.03). For the bond *J*_1_, all the light remains in the waveguide excited at the input, which indicates no transfer of light (Λ_1_=0). We also estimated the value of Λ_1_ and its fluctuations, which were both found to be insignificant, by analysing the exponential decay of evanescent coupling as a function of the inter-waveguide separation. The close proximity of these measured couplings to the aforementioned desired values suggests that anomalous edge modes should be detected for an input wavelength of 785 nm. Indeed, the quasienergy spectrum corresponding to these experimental values (Fig. 2c) is similar to the ideal case (Fig. 2b). While a finite dispersion of the bulk now becomes apparent, we note that the velocity of the edge modes remains significantly larger compared with the dispersion of the bulk. It should be noted that the non-zero standard deviations indicate that there will be bond-strength disorder within the lattice, while detailed numerical studies of the experimental results also suggest the additional presence of a small on-site (diagonal) disorder effect; the latter can be phenomenologically modelled by a small random on-site term. However, we verified that the topological properties of the lattice are unaffected by these sources of disorder (Supplementary Note 3).

### Observation of anomalous edge modes

**Figure 3:**
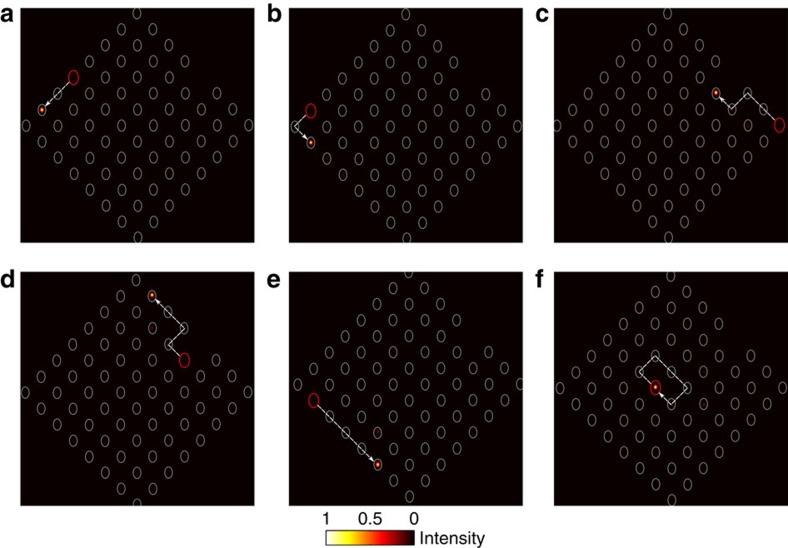
Experimental observation of anomalous topological edge modes. Experimentally measured output intensity distribution when the light is launched at the lattice site indicated by the red circle. The figures display chiral edge modes (**a**–**e**) that are not scattered by corners (**b**) nor defects (**c**,**d**) as well as a largely localized bulk state (**f**). The group velocity of the chiral edge modes along the bottom left edge of the lattice (**e**) is twice of that along the top left edge (**a**), which stems from the bond *J*_1_ having a different strength to the other three bonds.

**Figure 4:**
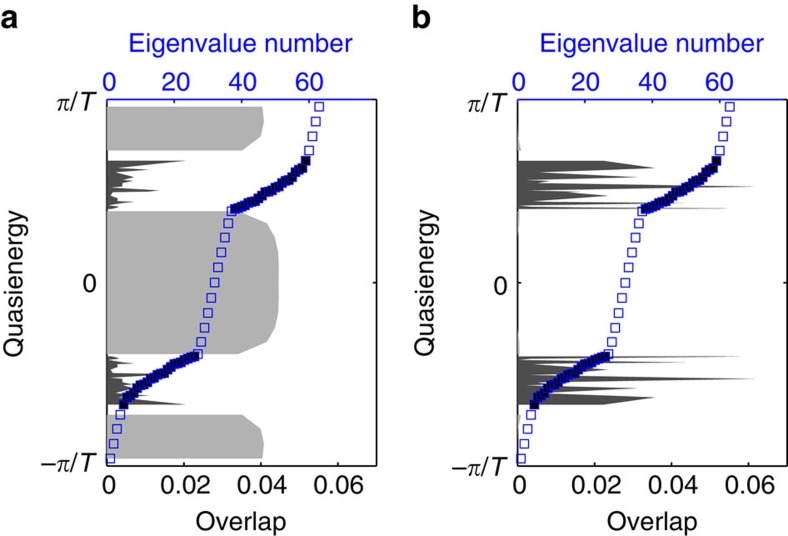
Quasienergy spectrum and the overlap. Numerically calculated quasienergy spectrum for the 63-site lattice and the overlap, as a function of this quasienergy, of two initial states with the Floquet eigenstates; these two initial states correspond to the light input of Fig. 3a,f, respectively. The filled blue squares correspond to the quasienergy of bulk Floquet states whilst the empty blue squares correspond to edge modes. The grey (resp. black) regions indicate the overlap with the edge (resp. bulk) states. (**a**) A single-site excitation on the edge of the lattice (excited site coordinates (4, 8)) predominately excites the topologically protected edge modes, which thereby yield the robust chiral motion that is observed in Fig. 3a. (**b**) The excitation of a bulk lattice site (excited site coordinates (4, 5)) almost entirely excites the two bulk bands, resulting in the bulk dynamics of Fig. 3f.

**Figure 5:**
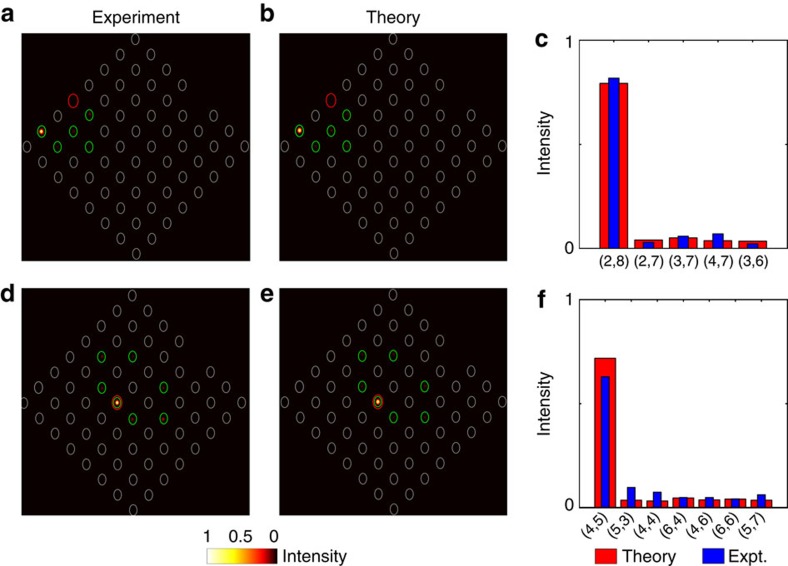
Comparison between experiment and theory. (**a**) Same result as in Fig. 3a, but using a different colour-scale so as to highlight the low intensities (bulk response). (**b**) Theoretical simulation of the intensity distribution, from a numerical resolution of the Schrödinger equation associated with the Floquet (effective) Hamiltonian. (**c**) Relative light intensity at the lattice sites where light was detected at the output (see green circles in **a**,**b**), showing the comparison between (**a**,**b**). Here the sites coordinates correspond to the axis orientation defined in Fig. 1f. (**d**) Same as Fig. 3f, and the corresponding simulated intensity distribution is shown in **e**. (**f**) Comparison between **d**,**e**.

To experimentally demonstrate the presence of anomalous topological edge modes, 785 nm light was launched at multiple locations around the edge of the lattice; the red circles in Fig. 3 indicate the launch site. If light is launched at the middle of an edge, as shown in Fig. 3a, then at the output it is observed that the light has moved along the edge with minimal penetration into the bulk. Moreover, if the input position of the beam is moved further down the edge, Fig. 3b, the close proximity of the launch site to the left edge means that the light will encounter the corner of the lattice during its evolution. This corner, as can be observed in the figure, does not cause backscattering but instead the light simply turns the corner and continues to propagate. This robustness is also observed in Fig. 3c,d, which demonstrates the light moving around a missing lattice site without backscattering or penetrating into the bulk. These observations provide evidence for the existence of protected chiral edge modes that can be almost exclusively excited by single-site excitation. These experimental observations are well complemented by a theoretical analysis, which was obtained using the couplings extracted from the bond characterization data. Figure 4 shows the quasienergy spectrum for our lattice, as well as the overlap, as a function of this quasienergy, of two different initial launch states with the numerically calculated Floquet eigenstates. Figure 3a–e demonstrates how launching in a single-edge site leads to an almost ideal excitation of the edge modes with little probability for bulk modes being excited (Fig. 4a), even when the launch site is close to a defect or corner. Note that the residual excitation of the bulk, predicted in Fig. 4a, was experimentally observed in Fig. 3a–e. To highlight this fact, we show the relative output intensities at various edge and bulk lattice sites in Fig. 5a–c, which shows good agreement with a theoretical simulation. The latter was performed by inserting the mean values of *J*_*i*_ extracted from the experiment into the effective Hamiltonian, and resolving the corresponding Schrödinger equation numerically.

The central result of these experimental images is that they show the existence of edge modes propagating in a chiral (unidirectional) manner, and which are not scattered by corners nor defects. The absence of scattering provides strong evidence that there is topological protection for these edge modes. From the bond-strength measurements, combined with our theoretical analysis of the corresponding model, we demonstrate that these propagating states correspond to anomalous topological edge modes.

The single-site excitation that is used in the experimental set-up can also be exploited to probe the different edge modes that are present in the lattice. Moving the input site onto the bottom left edge (Fig. 3e) shows the light propagating with twice the group velocity observed in Fig. 3a. This experimentally demonstrates that, when the bond *J*_1_ is different to the other three bonds, edge modes with different group velocities can be observed. The exact dispersion relations of the edge modes can be altered by modifying the strength of *J*_1_. The ability to tune the group velocity could present a valuable experimental tool for the future, which could allow, for instance, the investigation of how the non-scattering behaviour of edge modes is modified by the interplay between group velocity and non-linearity.

The behaviour observed when light is launched in the bulk of the lattice (Fig. 3f) is markedly different to what occurs on the edge with the input state being almost exactly reformed at the output facet. This refocusing is due to the beating between the two bulk bands that are separated by a bandgap of ∼*π*/*T*. In an ideal lattice, Λ_2_=*π*/2, the bulk bands would be dispersionless (Fig. 2b) and so the initial state would continue to reform every two driving cycles. In the experimental lattice, however, deviations of the bonds strengths from the ideal case cause the bands to become dispersive (Fig. 2c), which indicates that the initial state would eventually disperse in lattices of very long propagation lengths; see also Fig. 5d–f. These deviations could be addressed in future work by careful optimization of the experimental set-up, so as to allow a closer realization of the ideal model.

## Discussion

The ability to easily excite edge modes, with almost unit efficiency, and the possible co-existence of chiral edge modes with a dispersionless bulk, are two particularly interesting features of this slowly driven photonic system. These properties make this lattice a promising platform for investigating topological transport properties in response to perturbations, such as external (engineered) fields, disorder^31^ and particle–particle interaction (as generated by optical non-linearities).

We note that similar experimental results are reported in ref. 32.

## Methods

### Fabrication and characterization

The photonic lattice with two driving periods was fabricated inside a 70-mm-long glass (Corning Eagle^2000^) substrate using the ultrafast laser inscription technique, where the refractive index profile of each waveguide was controlled using the slit-beam shaping method^33^,^34^. The glass substrate, mounted on *x*–*y*–*z* translation stages, was translated at 8 mm s^−1^ through the focus of a 500 kHz train of 1,030 nm femtosecond laser pulses to fabricate each waveguide. The laser inscription parameters were optimized to produce waveguides that were single-mode and well confined in the measurement wavelength range of 700–830 nm. To study the response of the edge modes in the presence of a defect at the edge, the waveguide at the (8, 4) lattice site was not fabricated. It should also be highlighted that the waveguide paths are designed such that all waveguides exhibit identical bend radii at a given *z*, although the direction of this bending is site-dependent. This ensures that there is minimal site-dependent losses.

To measure the coupling constants, we fabricated five sets of each bond separately inside the same substrate. These bonds (or couplers) were then characterized in the wavelength range 705–795 nm, to obtain the variation of the mean and s.d. of coupling strength with wavelength (see Supplementary Fig. 3 for details). To excite the lattices with different wavelengths, a photonic crystal fibre^35^ was pumped by sub-picosecond laser pulses of 1,064 nm wavelength to generate a broadband supercontinuum. A tunable monochromator placed after the supercontinuum source was used to select narrow band (≈3 nm) light, which was coupled into an optical fibre (SMF-600). The fibre was then coupled to the lattice sites. The output intensity distribution was observed using a CMOS camera (Thorlabs DCC1545M). A polarizer passing only vertically polarized light is placed in front of the camera to ensure that the measurements are not affected by polarization-dependent coupling.

### Coupling between synchronously bent waveguides

In our experiment (Fig. 1e,f), we used synchronously bent waveguide pairs to turn the couplings on and off. In the Supplementary Note 1, we show that the coupling between two such bent waveguides is equivalent to an effective tight-binding coupling between two straight neighbouring waveguides. Hence, the propagation of light waves in our driven photonic lattice can be accurately described using a tight-binding model within the paraxial approximation.

### Computation of the topological phase diagram

In this section, the methodology used to obtain the topological phase diagram in Fig. 2 is detailed; this method is based on ref. 7, and we refer the reader to this reference for more details on concepts related to the topological characterization of Floquet systems. The calculation of the different phases is assisted by utilizing the property that the topology of the system can only change when there is a gap closing between the two bulk bands. The position of these gap-closing events can be found analytically by diagonalizing the evolution operator at the end of the driving period. It is found that for Λ_1_=Λ_2_ and Λ_2_=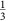
(2*π*−Λ_1_), the system is gapless at quasienergy zero, while for Λ_2_=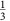
(*π*−Λ_1_) and Λ_2_=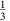
(3*π*−Λ_1_), the system is gapless through the fundamental zone edge. The position of these gap closings thereby divides the phase space into the eight different sectors shown in Fig. 2. The topology within these different sectors can then be defined by calculation of the winding numbers. As discussed in equation (2), at any time *t* the evolution operator may be diagonalized to yield the instantaneous Bloch bands of the driven system. The eigenstates associated with these bands can be used to calculate an instantaneous Chern number for that band. In accordance with equation (3), the winding number can then be calculated by tracking changes in the Chern number of the lowest band that occur through the zone edge throughout the driving period. This procedure is illustrated in Supplementary Fig. 2a for the parameters Λ_1_=1, Λ_2_=1.4, which shows how the instantaneous Chern number of the lowest band changes throughout the driving period. It can be readily observed that that the Chern number changes twice within a driving period and computing the spectra at these times shows that the second of these changes occurs through the zone edge (Supplementary Fig. 2b,c). This latter degeneracy causes the Chern number of the lowest band to decrease by one. This topological transition through the zone edge when combined with the Chern numbers of all the bands being zero at *t*=*T* implies, in accordance with equation (3), that both *W*_1_ and *W*_2_ are equal to one.

### Data availability

Raw experimental data are available through Heriot-Watt University PURE research data management system (DOI: 10.17861/702555b8-d1d4-4e4b-be88-e6a5b9794758).

## Additional information

**How to cite this article:** Mukherjee, S. *et al*. Experimental observation of anomalous topological edge modes in a slowly driven photonic lattice. *Nat. Commun.* **8,** 13918 doi: 10.1038/ncomms13918 (2017).

**Publisher's note:** Springer Nature remains neutral with regard to jurisdictional claims in published maps and institutional affiliations.

## Supplementary Material

Supplementary Information Supplementary Figures 1-5, Supplementary Notes 1-3 and Supplementary References
