# Supplementary material for: Experimental observation of anomalous topological edge modes in a slowly driven photonic lattice
Source: Nat Commun. 2017 Jan 4;8:13918. doi: 10.1038/ncomms13918 (PMC5216118; doi:10.1038/ncomms13918)
Supplement: Supplementary Information — Supplementary Figures 1-5, Supplementary Notes 1-3 and Supplementary References [file ncomms13918-s1.pdf]

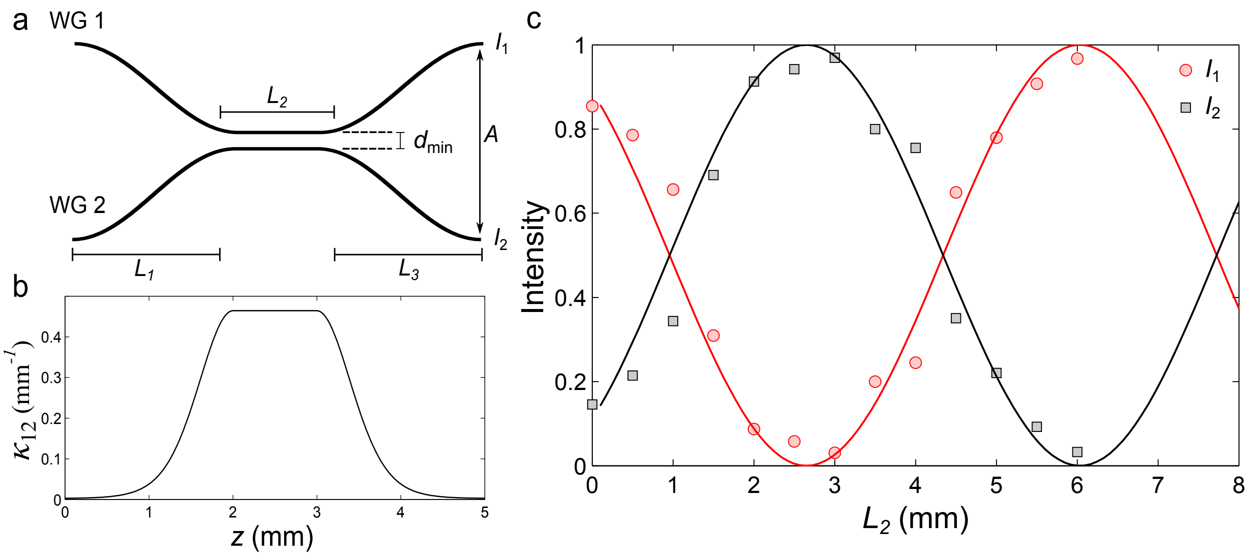

Supplementary Figure 1: **Characterisation of the directional couplers consisting of two synchronously bending waveguides.** (a) Schematic diagram of a directional coupler consisting of two synchronously bending waveguides, WG 1 and WG 2. The separation between the waveguides is $d(z)$, where z is the propagation direction. Here $d_{\text{min}} = \text{min}[d(z)]$ and $A = \text{max}[d(z)]$. (b) Variation of $\kappa_{12}(z)$ along the propagation direction of the coupler. (c) Experimentally measured (filled squares and filled circles) and theoretically predicted (solid lines) variation of intensities, (I_1, I_2), as a function of L_2.

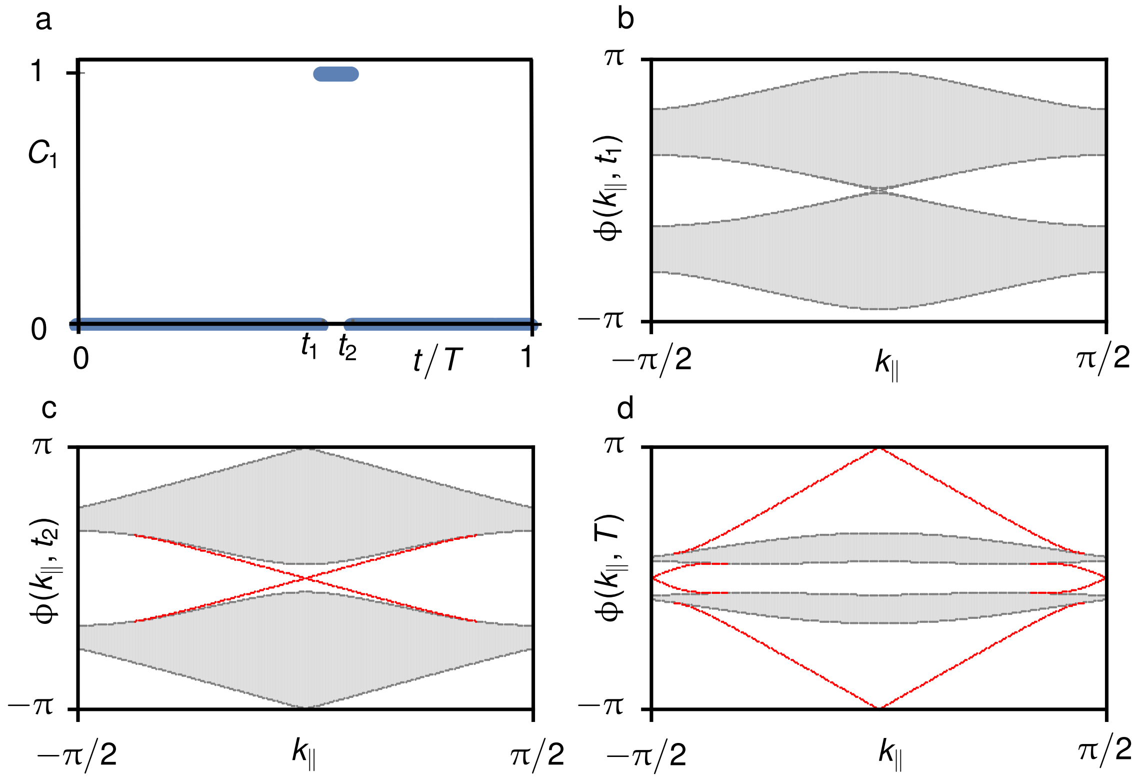

Supplementary Figure 2: **Example calculation of the winding number using the evolution of the instantaneous Chern number.** (a) Evolution of the instantaneous Chern number of the lowest band, C_1 over one driving period; the system parameters are $\Lambda_1 = 1$ and $\Lambda_2 = 1.4$. The Chern number changes twice within a driving period (at times t_1 and t_2). The first of these topological transitions occurs when the gap closes at $\phi = 0$, (b), whilst the second occurs at $\phi = \pi$, (c). This changing of the instantaneous Chern number that occurs through the zone edge is responsible for the winding numbers W_1 and W_2 being non-zero, and the Chern number of the Floquet bands to be zero. This non-trivial value for the winding number allows for the presence of edge modes in the spectrum at $t = T$, (d), even though the Chern numbers of all the Floquet bands (evaluated at $t = T$) are zero.

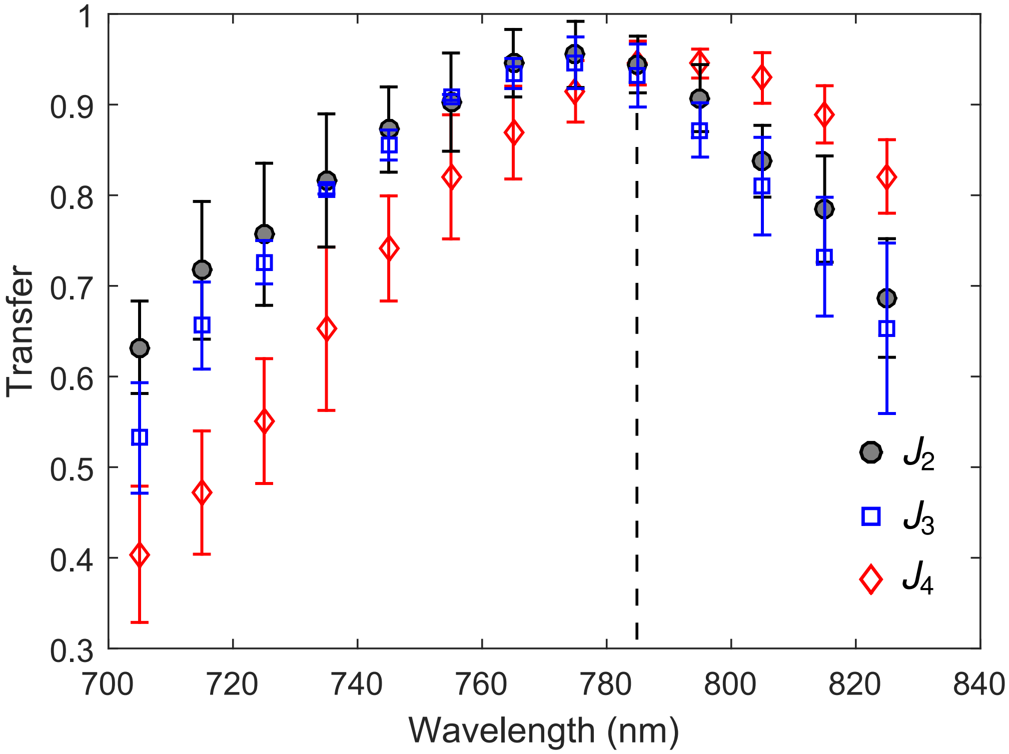

Supplementary Figure 3: **Variation of transfer for bonds** J_{2-4} **as a function of wavelength.** The effective coupling can be extracted from this characterisation data using the relation Transfer $= \sin^2(J_{\text{eff}}L)$, where L is the physical length of the sample. The error bars indicate the measured standard deviations. The dotted line indicates that the transfer is very close to 100% for all the three bonds at 785 nm wavelength. Note that the transfer for J_1 was measured to be zero.

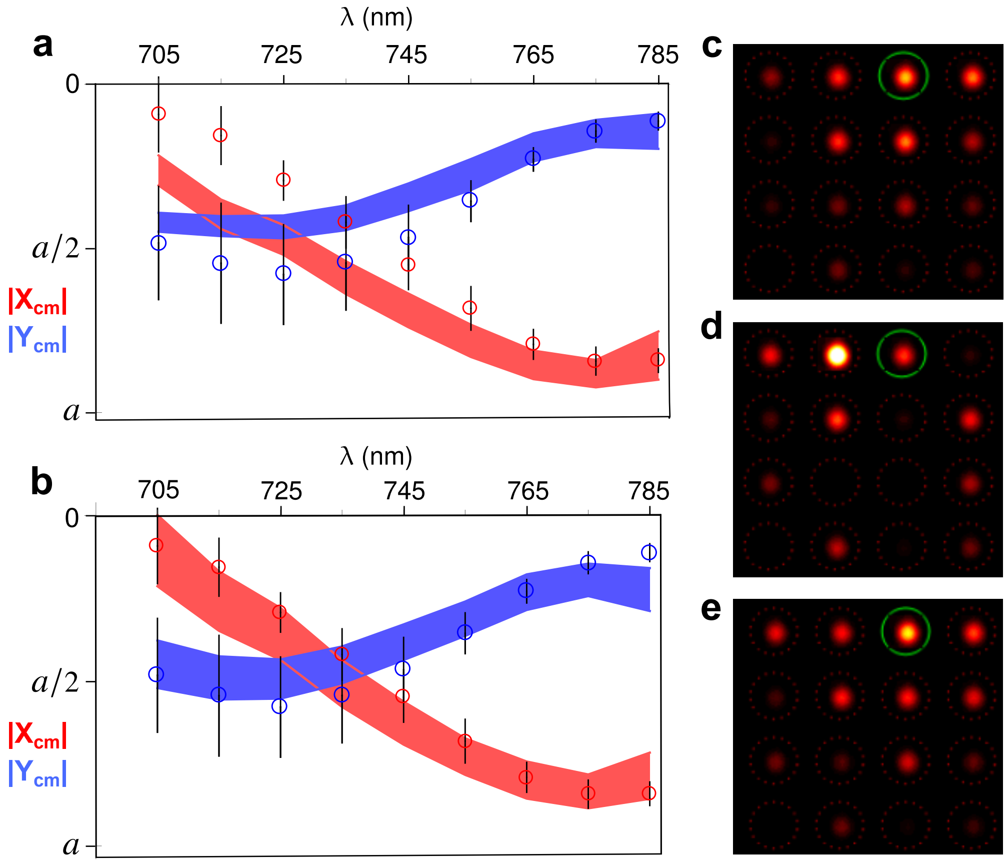
a
b
c
d
e
λ (nm)
705
725
745
765
785
0
a/2
a
|Xcm|
|Ycm|

Supplementary Figure 4 *(preceding page)*: **Comparing experimental results and theoretical predictions from two different disorder models.** (a) and (b) compare the experimental centre of mass drifts, as defined by Supplementary Equation (12), to those calculated theoretically for two models: model 1 includes bond strength disorder only whilst model 2 has additional on-site (diagonal) disorder. The bond strengths, J_i [$\Lambda_i = J_i T/4$], were randomly selected to lie within the range measured in the bond characterisation data in Supplementary Figure 3. The centre of mass drift for four different launch sites is measured experimentally and the red and blue circles indicate the average drift in the x and y directions whilst the black error bars indicate the measured standard deviations. The shaded regions in (a) and (b) indicate the theoretical disorder averaged value plus/minus one standard deviation. Note that in both panels (a) and (b), we considered the average over four lattice sites, two per opposite edges (the average being performed over the centre-of-mass drift in absolute value). (c) is the experimental output image, averaged over 4 launch sites, for an input light wavelength of 705 nm; here each data set has been translated so that the excited site corresponds to the one indicated by a green circle (in addition, data associated with the opposite edge have been rotated by 180 degrees). (d) and (e) are the disordered averaged output facet images obtained from models 1 and 2 respectively. In panels c-e, one shows 16 sites, located at the top edge of the lattice [here rotated by 45° with respect to Fig. 1 (f) in the main text].

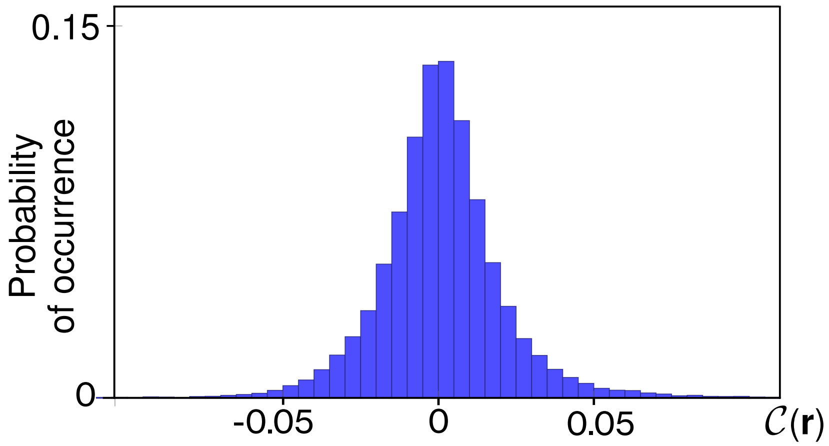

Supplementary Figure 5: **The local Chern marker for the experimental lattice with the addition of both diagonal and off-diagonal disorder terms.** The figure shows the probability of occurrence of a particular value for $\mathcal{C}(\mathbf{r})$ anywhere within the bulk of the lattice. This local Chern marker is narrowly peaked around zero which implies that the static bulk-edge correspondence would predict no robust chiral edge modes present in the system.

Supplementary Note 1: Coupling between bending waveguides

In the experimental setup, the lattice constant was chosen such that, in the absence of any bending, the waveguides comprising the lattice were practically uncoupled. In order to turn on the coupling between two neighbouring sites [see Fig. 1 (c) in the main text], the two waveguides were bent together and then remained straight before moving apart again [see Fig. 1 (e) in the main text]. The coupling between two such synchronously bending waveguides was demonstrated in ref. 1 to be equivalent to an effective coupling between two straight waveguides. This mapping is vital in order for the experiment to emulate our theoretical model, which assumes that the lattice is comprised of straight waveguides but with controllable couplings to neighbouring waveguides [see Fig. 1 (c) in the main text]. In light of the importance of this result in the present work, a derivation is provided here for completeness. For ultrafast laser inscribed optical waveguides, the refractive index contrast is small [$(n_{\text{eff}} - n_s) \ll n_s$; n_s and n_{eff} are the refractive index of the substrate and the effective refractive index of the guided fundamental mode respectively]. In this situation, the dynamics of how light propagates through an array of weakly-guiding waveguides is governed by the scalar Helmholtz equation,

$$\nabla^2 \psi + \frac{1}{\lambda\!\!\!^{-2}} n^2(x, y, z) \psi = 0.$$

Here $\psi(x, y, z)$ is the electric field amplitude, $\lambda\!\!\!^{-} = \frac{\lambda}{2\pi}$, where λ is the wavelength of the light in free space, and $n(x, y, z)$ is the refractive index profile in the domain of interest. In our photonic lattice, the waveguides bend slowly, and hence, the wave propagation is primarily along the z-axis. Under this condition, the electric field amplitude can be represented as a slowly varying complex

field amplitude and a fast oscillating wave, $\psi(x,y,z) = E(x,y,z)e^{i\frac{n_s}{\lambda}z}$. Substituting ψ into the Helmholtz equation and ignoring $\partial^2 E/\partial z^2$ (paraxial approximation) gives

$$i\lambda\frac{\partial E}{\partial z} = -\frac{\lambda^2}{2n_s}(\frac{\partial^2}{\partial x^2} + \frac{\partial^2}{\partial x^2})E + \frac{1}{2n_s}(n_s^2 - n^2)E. \tag{1}$$

The refractive index profile can be written as $n(x,y,z) = n_s + \Delta n(x,y,z)$. However, in laser-written waveguide arrays, the refractive index change caused by the laser is small and so $\frac{1}{2n_s}(n_s^2 - n^2) \approx -\Delta n(x,y,z) = V[x,y,z]$. Consider now an array of moving waveguides where $V[x,y,z]$ is given by

$$V[x,y,z] = \sum_k V_0^k = \sum_k V_0[x - x_k^0 - x_k(z), y - y_k^0].$$

Here $V_0(x,y)$ is the refractive index profile of a single isolated waveguide, the index k labels the different waveguides and $(x_k^0 + x_k(z), y_k^0)$ is the z-dependent position of the kth waveguide. In the regime where the waveguides are far apart, compared to the width of V_0, the electric field can be well approximated by a superposition of the fundamental modes of the different waveguides,

$$E(x,y,z) = \sum_k c_k(z)u[x - x_k(z), y]e^{-i\beta z},$$

where $u[x,y]$ is the fundamental mode of an isolated waveguide and satisfies the equation

$$-\beta u[x,y] = -\frac{\lambda^2}{2n_s}(\frac{\partial^2}{\partial x^2} + \frac{\partial^2}{\partial y^2})u[x,y] + V_0[x,y]u[x,y]. \tag{2}$$

Inserting the electric field expansion into Supplementary Equation (1) gives, after some rearrangement,

$$i\lambda\sum_k(\dot{c}_k u_k + c_k\frac{\partial u_k}{\partial x}(-\dot{x}_k)) = \sum_k c_k\left[-\frac{\lambda^2}{2n_s}\left(\frac{\partial^2}{\partial x^2} + \frac{\partial^2}{\partial y^2}\right)\right.$$
$$\left. + \beta + V_0^k\right]u_k + \sum_{n\neq k} V_0^n \sum_k c_k u_k, \tag{3}$$

where we have introduced the notation $u_k = u[x - x_k^0 - x_k(z), y - y_k^0]$. The expression in the square brackets is equal to zero as a result of Supplementary Equation (2). Multiplying by u_m and integrating transversely gives

$$i \sum_k (\dot{c}_k p_{m,k} + c_k f_{m,k}) = \sum_k c_k t_{m,k}, \tag{4}$$

where

$$\begin{aligned} p_{m,k} &= \int_{-\infty}^{\infty} \int_{-\infty}^{\infty} \mathrm{d}x \mathrm{d}y u_m u_k, \\ f_{m,k} &= -\dot{x}_k \int_{-\infty}^{\infty} \int_{-\infty}^{\infty} \mathrm{d}x \mathrm{d}y u_m \frac{\partial u_k}{\partial x}, \\ t_{m,k} &= \frac{1}{\lambda\!\!\!^{-}} \sum_{n \neq k} \int_{-\infty}^{\infty} \int_{-\infty}^{\infty} \mathrm{d}x \mathrm{d}y V_0^n u_m u_k. \end{aligned}$$

A number of pertinent statements can be made about these integrals:

$$\begin{aligned} p_{m,k} &= p_{k,m}, \\ f_{m,m} &= 0, \\ f_{m,k} &= -\dot{x}_k I_{m,k}, \\ f_{k,m} &= \dot{x}_m I_{m,k}, \\ I_{m,k} &= \int_{-\infty}^{\infty} \int_{-\infty}^{\infty} \mathrm{d}x \mathrm{d}y u_m \frac{\partial u_k}{\partial x}. \end{aligned}$$

These statements have the corollary that

$$\frac{\partial}{\partial z} p_{m,k} = \dot{p}_{m,k} = I_{m,k}(\dot{x}_m - \dot{x}_k)$$

As previously mentioned, in the absence of any bending there is negligible coupling between any of the waveguides. The consequence of this is that in the presence of bending, only pairs

of waveguides are coupled at any given moment. The coupled mode equations for this system can then be written as block diagonal matrices with each block comprising a 2×2 matrix. This simplifies the following to a study of a two waveguide coupler as the analysis will straightforwardly generalise to the full lattice. Therefore, if there are two waveguides, labelled 1 and 2, that are synchronously bending, such that $\dot{x}_1 = -\dot{x}_2$, then it can be readily seen that $\dot{p}_{1,2} = 2f_{1,2} = 2f_{2,1}$. The coupled-mode equations for this 2×2 block can therefore be written in matrix notation as

$$i(\mathbf{P}\dot{\mathbf{C}} + \frac{1}{2}\dot{\mathbf{P}}\mathbf{C}) = \mathbf{T}\mathbf{C}, \tag{5}$$

where the matrices **P** and **T** have the form

$$\mathbf{P} = \begin{pmatrix} 1 & X(z) \\ X(z) & 1 \end{pmatrix}; \quad \mathbf{T} = \begin{pmatrix} 0 & \kappa_{12}(z) \\ \kappa_{12}(z) & 0 \end{pmatrix}. \tag{6}$$

Here $X(z) = p_{1,2}(z)$ and $\kappa_{12}(z) = t_{1,2}(z)$. To proceed further we introduce a new set of variables by $\mathbf{C} = \mathbf{M}\mathbf{W}$, where **M** is chosen such that $\mathbf{M}^\dagger\mathbf{P}\mathbf{M} = \mathbf{I}$. This change of variable matrix can be written as

$$\mathbf{M} = \frac{1}{\sqrt{2}} \begin{pmatrix} \frac{1}{\sqrt{1+X(z)}} & -\frac{1}{\sqrt{1-X(z)}} \\ \frac{1}{\sqrt{1+X(z)}} & \frac{1}{\sqrt{1-X(z)}} \end{pmatrix}. \tag{7}$$

Using this change of variables and multiplying from the left by $\mathbf{M}^\dagger$ allows Supplementary Equation (5) to be rewritten as

$$i\dot{\mathbf{W}} = \mathbf{M}^\dagger\mathbf{T}\mathbf{M}\mathbf{W},$$

with $\mathbf{M}^\dagger\mathbf{T}\mathbf{M}$ taking the diagonal form

$$\mathbf{M}^\dagger\mathbf{T}\mathbf{M} = \begin{pmatrix} \frac{\kappa_{12}(z)}{1+X(z)} & 0 \\ 0 & \frac{\kappa_{12}(z)}{-1+X(z)} \end{pmatrix}.$$

The equation for $\dot{\mathbf{W}}$ can be straightforwardly solved to yield

$$\mathbf{W}(z) = \mathbf{T}(z)\mathbf{W}_0,$$

where $\mathbf{T}$ has the form

$$\begin{aligned}\mathbf{T}(z) &= \begin{pmatrix} \int_0^z \mathrm{d}z \frac{\kappa_{12}(z)}{1+X(z)} & 0 \\ 0 & \int_0^z \mathrm{d}z \frac{\kappa_{12}(z)}{-1+X(z)} \end{pmatrix} \\ &\approx \begin{pmatrix} \int_0^z \mathrm{d}z\kappa_{12}(z) & 0 \\ 0 & -\int_0^z \mathrm{d}z\kappa_{12}(z) \end{pmatrix}.\end{aligned}$$

In the second equality, the cross-power term, $X(z)$, has been neglected, which is a valid approximation as long as the waveguides remain far apart throughout the bending motion. A return to the original $\mathbf{C}$ variables can be made by inverting the non-singular matrix $\mathbf{M}$,

$$\mathbf{C}(z) = \mathbf{M}(z)\mathbf{T}(z)\mathbf{M}^{-1}(0)\mathbf{C}_0,$$

where $\mathbf{C}_0$ are the initial conditions.

The waveguides in the experimental lattice are coming together and then moving apart. Consequently, at the end of the bending section, $z = L$, the waveguides are at the same separation as at $z = 0$ and so $\mathbf{M}(L) = \mathbf{M}(0)$. This allows the matrix product $\mathbf{M}(L)\mathbf{U}(L)\mathbf{M}^{-1}(0)$ to take the form

$$\mathbf{M}(L)\mathbf{T}(L)\mathbf{M}^{-1}(0) = \mathbf{U}(L) = \begin{pmatrix} \cos(\phi) & -i\sin(\phi) \\ -i\sin(\phi) & \cos(\phi) \end{pmatrix}, \quad (8)$$

with

$$\phi = \int_0^L \mathrm{d}z\kappa_{12}(z). \quad (9)$$

For comparison, the evolution operator for two straight waveguides of length L is given by

$$\mathbf{U}_{Str}(L) = \begin{pmatrix} \cos(JL) & -i\sin(JL) \\ -i\sin(JL) & \cos(JL) \end{pmatrix}, \tag{10}$$

where J is the coupling between the waveguides. As can be readily observed, this has the same form as Supplementary Equation (8) if we write ϕ as $\phi = LJ_{\text{eff}}$ where J_{eff} is an effective coupling. Note that for such a directional coupler, if WG 1 is excited at the input [see Supplementary Figure 1], the normalized output intensities are given by

$$I_1 = \cos^2(\phi); \quad I_2 = \sin^2(\phi) \tag{11}$$

To experimentally verify the validity of Supplementary Equation (11), we fabricated thirteen sets of couplers with $L_1 = L_3 = 2$ mm, $A = 40$ μm, $d_{\text{min}} = 9.5$ μm and $0 \leq L_2 \leq 6$ mm; the bending profile of the two waveguides are shown in Supplementary Figure 1 (a). In the bending region, each waveguide bends as a sine-squared function. In Supplementary Figure 1 (c), the measured variation of output intensities, (I_1, I_2), are plotted as a function of L_2. To theoretically explain the measured variation of (I_1, I_2), we estimated $\kappa_{12}(z)$ in the following way. First, we experimentally measured how $\kappa_{12}(d)$ varies as a function of the separation, d, between two straight waveguides, and an exponentially decaying behaviour was observed [2]. From this, we then estimated the variation of $\kappa_{12}(z)$ as a function of z, as the separation, $d(z)$, between the two waveguides of the coupler is known. Supplementary Figure 1 (b) shows this variation for $L_2 = 1$ mm. Using Supplementary Equation (9) and (11), we plotted (I_1, I_2) as a function of L_2 [solid lines in Supplementary Figure 1 (c)] which is in excellent agreement with our experimental result. Supplementary Figure 1 (c) proves that Supplementary Equation (11), and hence, all the

approximations made to obtain this equation are valid for ultrafast laser inscribed directional couplers with slow bending.

This analysis has been conducted for two waveguides, but can be readily extended to the full lattice discussed in the main text. The experimental lattice is written such that only pairs of waveguides are coupled at any given moment. The **P** and **M** matrices therefore have block-diagonal matrix form with the blocks all having the form of Supplementary Equation (6).

Supplementary Note 2: Wavelength Tuning and Disorder

In the main text it was discussed how the experimental setup provides the ability to modify the wavelength of light used as an input. This ability allows the effective coupling between waveguides to be altered without modifying the lattice. In order to understand the behaviour of the bonds J_{2-4}, see main text, as a function of wavelength, five sets of isolated bonds were written and the percentage of light transferred from the launch waveguide as a function of wavelength was measured, Supplementary Figure 3. This bond characterisation data illustrates how at 785 nm the bonds J_{2-4} have almost equal transfer which is close to 100%. This wavelength therefore closely matches the desired parameters discussed in the main text of having three bonds equal and perfectly transferring.

The capability of this wavelength tuning technique in our experimental setup allows for the transfer of the bonds to be changed substantially from $\approx 100\%$ right down to $\approx 50\%$. This provides the ability to compare experimental and theoretical predictions for a wide range of parameters. In order to perform such a comparison, the coupling strengths extracted from the bond characterisation data were used to calculate a theoretical centre of mass drift, $\mathbf{r}_{cm}$, that could be compared to experimental results. The centre of mass drift is defined as

$$\mathbf{r}_{cm} = \sum_{m,n} I_{m,n}\mathbf{r}_{m,n} - \mathbf{r}_0, \tag{12}$$

where $I_{m,n}$ is the output intensity in the site (m,n), $\mathbf{r}_{m,n}$ is the spatial coordinate of the site (m,n), and $\mathbf{r}_0$ are the coordinates of the launch site. In the experiment, we separately excited the lattice sites of coordinates (4, 1), (6, 1), (4, 8) and (6, 8), which in the ideal (disorder-free) situation

[$\Lambda_1 = 0$, $\Lambda_{2,3,4} = \pi/2$], would correspond to only exciting the edge modes. By measuring the output intensity distributions after two driving periods, as a function of the incident-light wavelength, we calculated the centre-of-mass drift along the x and y directions.

These different positions should, in the absence of disorder, produce identical results for $\mathbf{r}_{cm}$, up to a sign change. However, the non-zero standard deviations measured in the bond characterisation data indicates the presence of bond strength disorder within the lattice (such a disorder will be referred to as off-diagonal disorder). The effects of this disorder can be directly observed in the experimental lattice with the four different launch sites producing slightly different results for $\mathbf{r}_{cm}$, see the error bars in Supplementary Figure 4 (a). Note that for the specific incident-light wavelength considered in the main text (785 nm), we find that $X_{\text{cm}} \approx -a$ and $Y_{\text{cm}} \approx 0$, which is in agreement with the edge-mode (chiral) displacement observed in Fig. 3(a) in the main text.

In order to theoretically model this disorder, the strengths of the bonds J_{2-4} were randomly selected to lie within the range measured in the bond characterisation data. The comparison between the experimental data and the theoretical prediction shows good agreement around $\lambda \approx 785$ nm, but deviations are observed at smaller wavelengths, Supplementary Figure 4 (a). The failure of the theoretical model in this low transfer region is further illustrated in Supplementary Figure 4 (c-e), which compares the averaged experimental output image to the theoretical image averaged over 1000 disorder realisations.

The match between theory and experimental results can be improved by including a small on-site disorder term, $\Delta\beta_{m,n}$, in addition to the coupling disorder of the previous model (this form

of disorder will be referred to as diagonal disorder). The $\Delta\beta_{m,n}$ for the (m, n) site is a random number drawn from within a uniform distribution covering the interval $[-W, \quad W]$. The parameter W is the disorder strength which is chosen as $WT/4 = 0.6$, as this particular value results in the best fit to the experimental data over the whole wavelength range investigated. The corresponding center-of-mass prediction, Supplementary Figure 4 (b), and output facet image, Supplementary Figure 4 (c), produced by this model are in closer agreement to the experimental results which points towards a disordering of this type being present in the lattice.

Supplementary Note 3: Study of Disorder and its impact on topological bands

The analysis conducted in the previous section illustrated the presence of off-diagonal disorder as well as possibly indicating the presence of diagonal disorder terms. In the absence of any disorder, the experimental lattice should reproduce a driven-lattice model that exhibits anomalous topological edge modes (i.e. chiral edge modes that are associated with non-zero winding numbers, while the Chern number of the Floquet bands is trivial; see main text). The inclusion of disorder in the system can allow bandgap closings to occur which can modify the topology of the system. In particular, the disorder could potentially change the Chern numbers of the Floquet bands to a non-trivial value such that the experimentally observed edge modes would no longer be anomalous. The elimination of this possibility thereby requires the calculation of the Chern number in a disordered system.

The presence of disorder means that quasimomentum is no longer a good quantum number which in turn precludes the use of the usual momentum space techniques in calculating the Chern number. In order to overcome this limitation we use a real-space Chern invariant, $C(\epsilon_0)$, as introduced by Bianco and Resta in ref. 3. This invariant generalises the ability of the *static* bulk-edge correspondence in predicting the number of edge modes in a band gap at energy ϵ_0 to also include disordered and quasi-periodic lattices [3,4]. In particular, when the energy, ϵ_0, is placed in a mobility gap of the spectrum the Chern invariant will measure the number of chiral edge modes which cross this energy, as predicted by the Chern numbers of the bands below this energy. This real-space Chern invariant is based around an operator called the Chern marker, $\hat{\mathcal{C}}$, which is

defined by

$$\hat{\mathcal{C}} = -4\pi \text{Im}[\hat{x}_Q \hat{y}_P]. \tag{13}$$

Here the operators $\hat{\mathbf{r}}_P = \hat{P}\hat{\mathbf{r}}\hat{Q}$ and $\hat{\mathbf{r}}_Q = \hat{Q}\hat{\mathbf{r}}\hat{P}$ are expressed in terms of the position operator $\hat{\mathbf{r}} = (\hat{x}, \hat{y})$ and the projection operators

$$\hat{P}(\epsilon_0) = \sum_{\epsilon \leq \epsilon_0} |\psi_\epsilon\rangle\langle\psi_\epsilon| = \hat{1} - \hat{Q}. \tag{14}$$

In the absence of disorder a real space Chern Number can be defined by averaging the Chern marker operator over a unit cell,

$$C = \int_{cell} \langle \mathbf{r}|\hat{\mathcal{C}}|\mathbf{r}\rangle d\mathbf{r} = \int_{cell} \mathcal{C}(\mathbf{r}) d\mathbf{r}. \tag{15}$$

In the presence of disorder this real-space Chern number fluctuates depending upon the position of the unit cell. These fluctuations can be accounted for by replacing the unit-cell average by an average over an area A that is located within the bulk and is large compared to the fluctuation length [4].

These ideas thereby provide a mechanism to investigate whether the disorder terms discussed in the previous section can alter the Chern numbers of the Floquet bands to non-trivial values. In particular we calculate the local Chern marker, in the presence of these disorder terms, for $\epsilon_0 = 0$. The real-space average of this local Chern marker, the Chern invariant, can be viewed as the prediction coming from the static bulk-edge correspondence for the number of chiral edge modes that are traversing the mobility gap between the two bands. The results, however, of this analysis reveal that even in the presence of disorder the local Chern marker, $\mathcal{C}(\mathbf{r})$, is still heavily peaked

around zero with this local quantity fluctuating only slightly within the bulk, Supplementary Figure 5. The Chern invariant is therefore equal to zero independently of the size of the averaging area that is chosen. Consequently, the static bulk-edge correspondence predicts the experimental lattice should not feature any chiral edge modes.

1. Huang, W. & Haus, H. A. Self-consistent vector coupled-mode theory for tapered optical waveguides. *Journal of Lightwave Technology* **8**, 922–926 (1990).

2. Szameit, A., Dreisow, F., Pertsch, T., Nolte, S. & Tünnermann, A. Control of directional evanescent coupling in fs laser written waveguides. *Optics Express* **15**, 1579–1587 (2007).

3. Bianco, R. & Resta, R. Mapping topological order in coordinate space. *Physical Review B* **84**, 241106 (2011).

4. Tran, D.-T., Dauphin, A., Goldman, N. & Gaspard, P. Topological hofstadter insulators in a two-dimensional quasicrystal. *Physical Review B* **91**, 085125 (2015).
